# Characterizing stress during animal interaction: a focus on the human endocrine response during equine-assisted services

**DOI:** 10.3389/fvets.2023.1303354

**Published:** 2023-12-18

**Authors:** Brandon R. Rigby

**Affiliations:** ^1^Exercise Physiology Laboratory, School of Health Promotion and Kinesiology, Texas Woman's University, Denton, TX, United States; ^2^Institute for Women's Health, Texas Woman's University, Denton, TX, United States

**Keywords:** equine-assisted activities and therapies, hippotherapy, horse physiology, neuroendocrine, rider physiology, therapeutic horseback riding

## Abstract

Repeated stresses applied to the rider may contribute to the documented physical and psychosocial outcomes from equine-assisted services. In this brief review, a summary of neuroendocrine markers of stress, including immunoglobulin A, serotonin, cortisol, progesterone, and oxytocin, is presented within the context of the physiology of stress modulation. Results are mixed with regard to the effects of these hormones on rider physiology before, during, and after equine-assisted services. However, some results from existing studies are promising with regard to the attenuation of stress. Future research should include a cross-disciplinary approach when conducting well-controlled studies with proper treatment and experimental fidelity, while also considering exogenous and endogenous factors that influence rider physiology.

## 1 Introduction

Equine-assisted services (EAS) include various services in which the horse and other equines are utilized to benefit individuals (1). These services include therapy, learning, and horsemanship (1). Previous terms used to denote therapy in this unique setting have included equine-assisted therapy and hippotherapy. Learning and horsemanship have been previously referred to as equine-assisted activities or therapeutic horseback riding. However, these latter terms have been recommended to be discontinued in favor of more inclusive language [for a more detailed summary and consensus on current terminology, see (2, 3)].

During EAS, the rhythmic movement of the horse is used as a tool to improve physical, emotional, and mental health in the rider (4). The physical (5) and psychosocial (6) outcomes of EAS in the rider have been summarized previously. While these exogenous effects have been characterized, the endogenous changes that may allow for improvements in overall function during EAS have not been summarized to date. More specifically, these changes include the stress applied to the rider. Stress is defined as the threat, or perceived threat, to an organism's homeostasis (7). Repeated stresses on bodily systems can lead to physiological adaptations and re-establishment of homeostasis, eliciting an improvement in functional capacity and physical function (7, 8). As the rhythmic movement of the horse elicits repeated bodily movements in the rider during EAS, it follows that the physical adaptations [e.g., gross motor function, balance, posture, muscle asymmetry, spasticity; (5)] to EAS occur, in part, due to the application of acute bouts of stress.

In humans, this adaptive stress response is influenced by cellular, molecular, and neuroendocrine factors located in the central nervous system and periphery (7, 9). When in a stressful state, a fast and slow response occurs (10). The fast response is mediated by the sympathoadrenomedullary (SAM) system, which promotes an increase in epinephrine and norepinephrine from the adrenal medulla and an additional increase in norepinephrine from the sympathetic nerves (10, 11). Once released, these hormones initiate the contraction of muscle cells in the vasculature, skeletal muscle, heart, and other organs (12, 13), resulting in increased physiologic responses that include, but are not limited to, vasoconstriction, blood pressure, heart rate, cardiac output, oxygen consumption, thermogenesis, arousal, alertness, and vigilance (10). The slow response is derived form the activation of the hypothalamic-pituitary-adrenal (HPA) axis, a critical structure both centrally and peripherally located to mediate the stress response (7, 10, 14). The activation of the HPA axis causes a release of corticotropin-releasing factor (CRF) from the hypothalamus in the brain (10). Interestingly, equines present with a similar neuroendocrine profile as humans under periods of stress. Both the SAM system and HPA axis also modulate the release of these hormones in equines (15, 16).

Changes in neuroendocrine responses can attenuate stress in humans following animal interaction (17). Other endogenous markers that have been positively affected in the rider following human-animal interaction include serotonin (18), immunoglobulin A (19), and oxytocin (20). In this brief review, a summary of the neuroendocrine markers of stress is presented. Concentration changes before, during, and after EAS are provided. A brief summary of the stress incurred by the equine during EAS is also presented. Finally, future directions for research are discussed.

## 2 Immunoglobulin A

Immunoglobulin A (IgA) is the most dominant antibody in immunity, regulating mucosal homeostasis (21). Once secreted in the lumen, it binds to antigens and prevents toxins from entering the submucosa and circulation (22). Acting as the primary barrier to pathogens and irritants, increased concentrations may promote immune health (21). As the immune system is integrated with other organs in the body, including the brain, behavior and activity (including tactile contact between mammals) can positively affect immunity (23). Additional lifestyle habits may increase concentrations of IgA, including listening to music (24), relaxing (25), or watching a funny movie (26). Markers of immunity and the associated physiological stress response have remained mostly unexplored in the interaction between humans and equines. However, concentrations of IgA did not change after 15 min of equine-facilitated learning (i.e., moving around a horse while focusing on the response of the horse with awareness of their own bodily sensations) in healthy older adults (27).

## 3 Serotonin

Serotonin, a neurotransmitter in the central nervous system, is implicated in the function of the cardiovascular, pulmonary, metabolic, gastrointestinal, and genitourinary systems (28). The influence of serotonin on gastrointestinal function and gut microbiome is great, as more than 90% of circulating serotonin in the periphery is synthesized by cells in the gastrointestinal tract (29). Metabolically, it contributes to glucose homeostasis and adiposity, thus influencing the course and progression of chronic diseases (29).

Serotonin receptors are expressed in many regions of the brain, and thus help to regulate the nuclei in these regions involved in behavioral output. In addition to well-known attributes such as mood, sleep and appetite, there are multiple behavioral attributes that are regulated by serotonin, including perception, reward, anger, aggression, memory, and attention (28). More specifically, serotonin may play a key role in emotional regulation and behavioral flexibility, social cognition and control of social interactions, anxiety, and learning and memory (30, 31). The influence of serotonin may therefore be very important to quantify before and after the administration of EAS. There are no known studies that include the measurement of serotonin following an acute or long-term EAS intervention in chronic disease populations. However, serotonin concentrations increased by 5% following 8 weeks of horseback riding in a therapeutic setting in older adults (32).

## 4 Cortisol

When secreted from the hypothalamus, CRF stimulates the nearby anterior pituitary gland to release adrenocorticotropin (ACTH), eliciting the release of cortisol from the adrenal cortex into the circulation (33). Its metabolic effects include lipolysis, protein catabolism, and an increase in blood glucose by stimulating liver enzymes, although this glucose is blocked when delivered to working tissues (34). However, this mobilization of energy is thought to be the primary function of cortisol during periods of stress (35).

Cortisol responses to EAS have been mixed. Morning salivary cortisol concentrations did not change over the course of 6 weeks of equine therapy in veterans with PTSD (36), or after 10 weeks of therapeutic horseback riding in children with autism spectrum disorder [ASD; (37)]. However, a 20 and 24% decrease in salivary cortisol concentrations were observed after 1 month (38) and 12 weeks (39) of weekly hippotherapy sessions, respectively in male children with ASD. There are positive results reported in healthy populations. Cortisol concentrations decreased by 6% following 8 weeks of horseback riding in a therapeutic setting in older adults (32). In addition, cortisol levels were decreased in these participants when compared to a control group following the riding protocol (32). Finally, 11 weeks of equine-facilitated activities performed once per week, 90 min per session, decreased salivary cortisol concentrations by 20% in healthy adolescents (40). In healthy adults without horseback riding experience, no change in cortisol concentrations was observed in healthy adults following a 2-h horse-riding lesson program (41). However, among healthy adults with horseback riding experience, a 61% and 64% decrease in cortisol concentrations were observed immediately following, and 1 h following, a 2-h horse-riding lesson program, respectively (41). The observed decrease in cortisol concentrations following EAS may be expected, as many of those who participate in these interventions experience relaxing effects during the treatment (42).

## 5 Progesterone

Traditionally thought of as a female sex hormone, progesterone is produced in the corpus luteum in the ovaries and metabolized primarily in the liver in women (43). In men, progesterone is secreted by the adrenal cortex, and can be metabolized to other sex hormones, including testosterone and estradiol (44). Progesterone also influences mood and behavior via emotion processing (43). Inclusive to emotion processing are emotion recognition accuracy and emotional memories (45). When progesterone concentrations are high, there is a faster response to negative stimuli, which typically presents as a heightened sensitivity to physical threats (46–49). Emotional memories are mediated by the HPA axis, and elevated progesterone levels are correlated with emotional free-recall and recognition memory (50, 51). To date, these phenomena have been only observed in women. However, due to the influence of progesterone on the HPA axis and the brain, the hormone may have positive effects on mood, cognition, and neuronal growth in men and women (52–54).

Acute assessments of progesterone have been completed in male children with ASD after one session of hippotherapy. An increase of 80% in salivary progesterone was observed after 30 min of therapy (38). Longer-term assessments have also been made, with a 21% and an 83% increase in salivary progesterone after 1 month (38) and 12 weeks (39) of weekly hippotherapy sessions, respectively, in the same population. Based on these results, the regulation of mood may be enhanced with EAS in children with ASD due, in part, to the release of progesterone derived from the rider's motivation to bond with the horse (55).

## 6 Oxytocin

Oxytocin is primarily synthesized in the hypothalamus and released from the pituitary gland (56). Once activated, its presence in the blood affects other organs, including the mammary glands and kidneys (57). Oxytocin may therefore play a crucial role with stress-related behaviors due to its influence within the HPA axis (57). More specifically, the release of oxytocin may elicit decreases in glucocorticoids and concomitant increases in parasympathetic nervous system function, thereby decreasing heart rate and blood pressure (BP) responses (17, 58). Indeed, the active form of oxytocin is related to reduced anxiety and relaxation in children (59). Oxytocin is also implicated in the underlying mechanisms of the development and maintenance of attachment in mammals (60). More specifically, tactile contacts (e.g., touch, warmth, vibration), which are critical for social bonding, may be facilitated by oxytocin (61). The human-animal interaction and, specifically, tactile contact, stimulates the release of salivary, plasma, and urinary oxytocin (62, 63). The validation of salivary oxytocin has recently been shown in mammals (62, 64).

## 7 Other stress-related measures

Cardiopulmonary measures, including heart rate, BP, pulmonary function, and heart rate variability (HRV), have been characterized as stress-related markers during EAS. Overall, hippotherapy and EAT does not alter heart rate in children with neurologic disorders (65–67) or adults (68), respectively. However, heart rate responses may change based on the level of disability of the rider. Indeed, therapeutic horseback riding may elicit increases in heart rate in children with a moderate to severe pathophysiology (69), particularly when compared to youth with less motor impairment (70). Heart rate responses are lower when riders are grooming and petting horses when compared to walking and leading them (71). A single session of, or training over time involving, EAT or hippotherapy also does not alter BP (65–67, 71) or respiratory responses (67, 69, 71) when assessed after sessions have ended vs. baseline. Fifteen minutes of equine-assisted learning may, however, increase respiratory responses in older adults (27). Finally, based on HRV measures during EAS, there may be an attenuation of sympathetic nervous system activity and an increase in parasympathetic nervous system activity [see (72), for a comprehensive review], thereby resetting balance during periods of perceived stress, and promoting improvements in cognitive and emotional control (73, 74).

## 8 Considerations of the horse

The stress level of the horse should be considered to ensure the health and welfare of these animals are maintained (75). Because horses are prey animals, they have a heightened awareness to their environment, unlike dogs or cats (36). As such, they are able to perceive, respond, and learn from subtle stimuli in a therapeutic setting (76). Equines can respond to a human presence through changes in the rider's physiology, body language, and vocal tones (76). This response may be accompanied by acute stress in the horse, either from the rider or from another environmental stimuli. Confusion and related conflict behaviors in the horse, when accompanied by stress, can then lead to injury to horse riders and handlers and perpetuate the idea that horses exhibit unpredictable behavior (77). Indeed, if the rider is stressed, this may increase the likelihood of a startled reaction in horses via a transmission of stress, thus increasing the risk of injury (78). Elevated stress levels can elicit negative effects on immunity in horses, which may lead to an increased incidence of colic and gastric ulcers (79–81). Also, strong emotional reactions may impair learning performance in equines (82–84). Taken together, these effects may decrease equine performance and attenuate the benefits received by the rider during EAS.

Although stress responses can be variable, the therapy setting typically does not add stress to the horse. No difference in cortisol concentrations was found between modes of riding [i.e., therapeutic vs. traditional; (85)] or between time points within and across EAS sessions (41, 71). This is significant, as serum-free cortisol concentrations increase within 10 min after exposure to acute stress in equines (86). Furthermore, cortisol, ACTH, and glucose maintained normal ranges over time, and between riders with and without posttraumatic stress disorder (75). Alternatively, a decrease in cortisol levels in the horse by up to 45% may occur when children with psychomotor disabilities participate in EAT (87).

Stress can also be objectively measured in horses using cardiovascular measures, including HRV. Similar to measurements in humans, HRV measures in horses are sensitive and reliable indicators of fear or anxiety (27). Basal HRV variations can be influenced by genotype, behavior, environment, temperament, and dietary habits (88). During a 15-min equine-facilitated learning session with older adults, very low frequency (VLF) ranges of HRV were recorded (27). Power within the VLF band is consistent with improved health (89), with the activity of the parasympathetic nervous system contributing most to VLF power (90). In another study, no changes in HRV were found in horses after several phases and sessions of EAT with riders who had varying health statuses (68, 71, 91). However, HRV measures were different between horses who performed equine-assisted activities and therapies and horses who performed dressage, jumping, and eventing activities (92).

## 9 Conclusion and future directions

In this review, IgA, serotonin, cortisol, progesterone, and oxytocin were summarized in the context of rider physiology during EAS. Although the characterization of the concentrations of these hormones is mixed throughout the literature, results from existing studies are promising with regard to the attenuation of stress in the rider during EAS. The positive outcomes in stress modulation may contribute to the physical and psychosocial benefits observed across populations of varying age, diagnoses, and horseback riding experience. However, it appears that periods of chronic stress, physiologically manifesting as high concentrations of glucocorticoids and catecholamines, can influence the ability for horses to perform during EAS and can negatively impact the human-animal bond.

Due to the cross-disciplinary nature of this topic, and the difficulty in obtaining and analyzing blood and saliva samples, future research teams should include biologists, biochemists, or some healthcare practitioner (e.g., nurse, phlebotomist, exercise physiologist). More well-controlled studies with proper treatment and experimental fidelity to allow for accurate quantification of stress-related hormone concentrations are needed in this area. A number of physiological factors in the rider should be considered, including method of data collection (salivary vs. plasma and/or serum), time of day (i.e., circadian rhythm), timing of data collection before, during, and after EAS sessions based on knowledge of peak hormone concentrations, and unique sex characteristics (e.g., male vs. female, timing of female menstrual cycle), as any of these can influence neuroendocrine responses to stress (93–95). With these future directions, the next steps can be made in the process of seeking to make EAS more affordable and accessible.

## Author contributions

BR: Writing—original draft, Writing—review & editing.
